# Fermented Papaya Preparation Restores Age-Related Reductions in Peripheral Blood Mononuclear Cell Cytolytic Activity in Tube-Fed Patients

**DOI:** 10.1371/journal.pone.0169240

**Published:** 2017-01-06

**Authors:** Yuhzo Fujita, Haruo Tsuno, Jiro Nakayama

**Affiliations:** 1 Yuno Onsen Hospital, Yuno, Syunan-shi, Yamaguchi, Japan; 2 Laboratory of Microbial Technology, Division of Systems Bioengineering, Department of Bioscience and Biotechnology, Faculty of Agriculture, Graduate School, Kyushu University, Hakozaki, Higashi-ku, Fukuoka, Japan; University of Sydney, AUSTRALIA

## Abstract

Tube-fed elderly patients are generally supplied with the same type of nutrition over long periods, resulting in an increased risk for micronutrient deficiencies. Dietary polyphenols promote immunity and have anti-inflammatory, anti-carcinogenic, and anti-oxidative properties. *Carica papaya* Linn. is rich in several polyphenols; however, these polyphenols are poorly absorbed from the digestive tract in their original polymerized form. Therefore, we determined the molecular components of a fermented *Carica papaya* Linn. preparation, as well as its effects on immunity and the composition of gut microbiota in tube-fed patients. Different doses of the fermented *C*. *papaya* L. preparation were administered to three groups of tube-fed patients for 30 days. Its effects on fecal microbiota composition and immunity were assessed by 16S rRNA gene sequencing and immune-marker analysis, respectively. The chemical composition of the fermented *C*. *papaya* L. preparation was analyzed by capillary electrophoresis- and liquid chromatography- time of flight mass spectrometry. The fermented *C*. *papaya* L. preparation restored peripheral blood mononuclear cell (PBMC) cytolytic activity; however, no other biomarkers of immunity were observed. Treatment with the preparation (9 g/day) significantly reduced the abundance of Firmicutes in the fecal microbiota. In particular, treatment reduced *Clostridium scindens* and *Eggerthella lenta* in most patients receiving 9 g/day. Chemical analysis identified low-molecular-weight phenolic acids as polyphenol metabolites; however, no polymerized, large-molecular-weight molecules were detected. Our study indicates that elderly patients who are tube-fed over the long-term have decreased PBMC cytolytic activity. In addition, low-molecular-weight polyphenol metabolites fermented from polymerized polyphenols restore PBMC cytolytic activity and modulate the composition of gut microbiota in tube-fed patients.

## Introduction

Tube feeding is recommended for patients with swallowing dysfunction and dysphagia associated with conscious disturbances and neural dysfunction, such as cerebrovascular diseases, severe head trauma, and advanced stage neurodegenerative diseases (e.g., Alzheimer’s and Parkinson’s disease). Tube-fed patients are prone to various types of complications such as infectious diseases, particularly of the respiratory system [1–5].

Immunity is regulated by the thymus gland, and thymus gland function is known to gradually decrease with age [6–11]; therefore, the immune system is generally weaker in older patients than in younger patients. Large mononuclear lymphocytes, known as natural killer (NK) cells because of their spontaneous killing of cancer cells and antiviral properties [12–14], are a component of adaptive immunity; it is well known that they exhibit reduced function and altered composition with aging [12,15].

Patients requiring tube feeding are typically provided with a variety of nutrients such as milk proteins, glucose, minerals (Na, K, Mg, Fe, and Zn), vitamins (B1, B2, B12, and E), and polyunsaturated fatty acids. However, there is little or no information regarding the effects of supplementation nutrients on the maintenance and promotion of host immunity to prevent infections and malignancies. Polyphenols are well-known anti-inflammatory and immune-modulatory agents [16,17] that are easily consumed from vegetables, fruits, wheat, tea, and beverages. Elderly patients can maintain immunity by consuming polyphenol-rich fruits and vegetables [18–25].

Commercially available tube-feeding formulas are primarily designed to supply standardized calories, but there is little information on their role in promoting immunity. Tube-fed patients often receive the same nutrients over a long period without daily or seasonal changes. We suggest that altering the micronutrients supplied on a daily basis can improve the immunity of tube-fed patients and prevent infections or malignancies.

*Carica papaya* L. is traditional medicinal plant rich in many types of polyphenols [26–30]. For example, the papaya fruit exocarp is rich in ferulic acid, caffeic acid, caffeoyl-hexoside, rutin, and quercetin 3-*O*-rutinoside. Furthermore, the mesocarp contains caffeic acid, caffeoyl-hexoside gallic acid, gallic acid hexoside, protocatechuic acid, protocatechuic acid hexoside, quercetin, myricetin, isorhamnetin, and kaempferol [26].

Fermented papaya preparations (FPPs) have been reported to have anti-inflammatory and immune-modulatory properties in both *in vivo* and *in vitro* experiments [31–34].

FPPs are the products of wild unripe *C*. *Papaya L*. fermented by *Enterococcus faecalis* and *Aspergillus oryzae*. Therefore, FPPs can be considered symbiotic, including both prebiotics and probiotics. Several studies have indicated that live bacteria in probiotics activate human immunity by stimulating the immune system in the colon [35–38].

In the present study, we examined the effects of FPP supplementation for 30 days on immunological and metabolic functions and fecal flora in tube-fed patients; the composition of FPP was also determined by chemical analysis. The results show that FPP enhance NK cell cytotoxicity and decrease the number of *Clostridium scindens* and *Eggerthella lenta* in the gut flora of elderly tube-fed patients.

## Materials and Methods

### Materials

FPPs (SAIDO-PS501, Lot No. S-50330) were obtained from SAIDO Co., Ltd. (Fukuoka, Japan). The juice from wild, whole, unripe, ground, *C*. *papaya* L. (including fruit skins and seeds) was extracted in the Philippines and exported to Japan where it was fermented for approximately one year with *E*. *faecalis*, followed by approximately six months with *A*. *oryzae*, and then dried. Both fermentation processes were conducted at room temperature under aerobic conditions.

### Chemical Analysis of FPP Components

The different FPP components were analyzed by capillary electrophoresis-time-of-flight mass spectrometry (CE-TOFMS) and liquid chromatography (LC-TOFMS) from 1 to 5 years after extraction at Human Metabolome Technologies, Inc. (Yamagata, Japan).

For CE-TOFMS measurements, approximately 100 mg of FPP was plunged into 500 μL of methanol containing an internal standard solution (50 μM; #H3304-1002; HMTI) at 0°C. FPPs were then homogenized thrice at 1,500 rpm for 120 sec using a tissue homogenizer (BMS-M10N21; Bio Medical Science Inc. BMS Tokyo, Japan). Then, 200 μL of Milli-Q water and 500 μL of chloroform were added to the samples, thoroughly mixed, and centrifuged for 5 min at 2,300 × *g* and 4°C. The upper aqueous layer (400 μL) was centrifugally filtered through a Millipore 5 kDa-cutoff filter to remove the proteins. The filtrate was then lyophilized and suspended in 50 μL of Milli-Q water and analyzed by CE-TOFMS.

For LC-TOFMS measurements, approximately 100 mg of FPP was plunged into 500 μL of methanol containing an internal standard solution (20 μM; #H3304-1002; HMTI) at 0°C to inactivate enzymes. The tissue was homogenized twice at 1,500 rpm for 120 sec using a tissue homogenizer (BMS·M10N21). The mixture was centrifuged at 2,300 × *g* at 4°C for 5 min. The supernatant was desiccated and then dissolved with 100 μL of 50% isopropanol /Milli-Q water for LC-TOFMS analysis at Human Metabolome Technologies, Inc.

The CE and -LC-TOFMS data were analyzed by Human Metabolome Technologies, Inc., using Master Hands automatic-integral analysis software (ver. 2.9.0.9, Keio University, Tsuruoka, Japan).

The mass-to-change ratio (*m/z*), migration time (MT), and relative peak area were obtained from the peak values. The relative peak area was computed with the following equation: Peak area ratio = targeted peak area/internal standard peak area.

### Clinical Study

#### Patients

Study participants included 8 males (mean age 77 ± 10.6, range 67–96 years) and 12 females (mean age 89 ± 5.8, range 77–97) patients with cerebrovascular disease (n = 12, 4 males and 8 females), neurodegenerative disease (n = 7, 4 males and 3 females), and post-traumatic head injury (n = 1, male). Study participants had been tube-fed for 1–7 years. The study protocol was approved by the human ethics committees of Yuno Onsen Hospital, and written consent for the study was obtained from a family member of each patient.

#### FPP treatment

Study participants were divided into three groups, including a control group that did not receive FPP (n = 5); the remaining two groups were administered 3 g FPP/day (n = 7) or 9 g FPP/day (n = 8). Treatments were administered once (3 grams) per day or 3 times (3 grams each) per day for 30 consecutive days.

#### Laboratory examination

Blood samples were collected and sent in cooling boxes within 24 h to BioMedical Laboratory (BML, Tokyo, Japan) for analysis. Samples were analyzed for peripheral blood mononuclear cell (PBMC) count, total serum protein, serum albumin, serum globulin, and liver function (total bilirubin, glutamic oxaloacetic transaminase (GOT), guanosine triphosphatase (GPT), gamma glutamyl-transpeptidase (γGTP), alkaline phosphatase (ALP), cholinesterase, leucine aminopeptidase (LAP), and lactic acid dehydrogenase (LDH)). In addition, renal function (blood urea nitrogen (BUN), creatinine, and uric acid) and lipid metabolites (total cholesterol (T-ch), high density lipoprotein (HDL), low density lipoprotein (LDL), and triglycerides (TG)) were studied using Roche Reflotron system kits (Roche Diagnostics Co., Ltd.). Serum electrolytes, as well as cellular and humoral immunity were additionally analyzed. IgG, IgA, and IgM were assayed using immunoglobulin kits from NITTOBO Medical (Koriyama, Japan); interleukins (IL-2, IL-6, and IL-10) and chemokines (interferon γ (IFN-γ) and tissue necrosis factor α (TNF-α)) were assayed by a quantitative enzyme immunoassay technique using a MXL (Dynex Technologies, Chantilly, VA, USA).

#### Cell isolation

PBMCs were isolated from peripheral blood (diluted 1.6 times with 10% phosphate buffered saline) by density gradient centrifugation (800 × *g* for 25 min at room temperature) over Isolymph (specific gravity 1.077) (CT Scientific Supply Corp., Deer Park, NY, USA) in Leucosep tubes (Greiner Bio-One, Tokyo Japan) for use in the NK cell activity assay. The supernatant (isolated PBMCs) was washed twice with saline solution and the efficiency of cell separation was confirmed by flow-cytometry (Accuri C6, Becton Dickinson and -Company, Franklin Lakes, NJ, USA).

#### Cytotoxicity assay

A ^51^Cr release assay was used to measure PBMC cytolytic activity; target cells (K562) were labeled with 100 μCi ^51^CrO_4_ (Perkin Elmer, Japan) for 60 min at 37°C in atmosphere of 5% CO_2_ in air. Labeled K562 target cells were adjusted to 2 x 10^5^ cells/mL in RPMI-1640 medium supplemented with 10% fetal bovine serum. K562 (1 x 10^5^ cells/well) and effector cells (1 x 10^4^ PBMC/well) at 1:10 and 1:20 effector/target (E: T) ratios were co-incubated in 200 μL of RPMI-1640 in 96-well U-bottomed plates in triplicate for 4 h at 37°C and 5% CO_2_ in air. Radioactivity was measured with a gamma scintillation counter (Perkin Elmer, Japan). The percentage of cytotoxic activity was calculated using the following formula:

specific lysis (%) = (sample cpm–spontaneous cpm)/(maximal cpm–spontaneous cpm) * 100 Large mononuclear lymphocytes have cytolytic activity against malignant tumor cells. The cytolytic activity of PBMCs against malignant cells such as K564 depends almost entirely on NK cell cytotoxicity [39]; therefore, we expressed NK cell cytotoxicity as cytolytic activity of PBMCs.

### Fecal Flora Analysis

Fecal bacterial composition was analyzed before and after administration of FPP and in the control group by high-throughput sequencing of 16S rRNA gene fragments amplified from each stool sample [40]. Briefly, whole genomic DNA was extracted from the stool samples by the bead-beating method [41] and the V6–V8 variable region of the 16S rRNA gene was amplified by PCR with universal primers Q-968F and Q-1390R, each carrying a barcode label sequence. The amplified fragments were mixed and applied to the pyrotag sequence using Roche 454 GS FLX Titanium system. The obtained sequences were processed by the QIIME 1.7.0. pipeline [42] equipped with USEARCH ver. 5.2.236 [43,44] for barcode splitting, denoising, chimera removal, and construction of operational taxonomic units (OTUs). Consequently, 568 OTUs, comprising 182,834 reads (mean ds/sample 3975 ± 680, minimum = 2117), were considered to be a non-redundant set of OTUs. The reads were subsampled for adjustment to 2,000 reads/sample. The taxonomy of each OTU was assigned using the RDP classification in the QIIME pipeline based on the Greengenes taxonomy (97 OTU taxonomy) and the Greengenes reference database (97 OTUs, FASTA) [45]. To search for closest species, the representative sequence of each OTU was subjected to RDP Seqmatch [46] in the Ribosomal Database Project II (http://rdp.cme.msu.edu/seqmatch/seqmatch.intro.jsp), in which the lower threshold of the S_*ab*_ score was set to 0.84. If more than two species showed the same highest scores, the one with the highest count among the top 20 matches was selected for annotating the species by using a Microsoft Excel macro file named Seqmatch Q400 [47]. The bacterial composition of each fecal sample was determined at each taxonomic rank according to the OTU table and the taxonomic information of each OTU.

### Statistical Analysis

All data are expressed as the mean ± SD. Differences between groups were compared using the Student’s *t*-test or analysis of variance (ANOVA). Differences were considered significant when *P* < 0.05.

## Results

### FPP Stimulates NK Cell Activity

**Fig 1:**
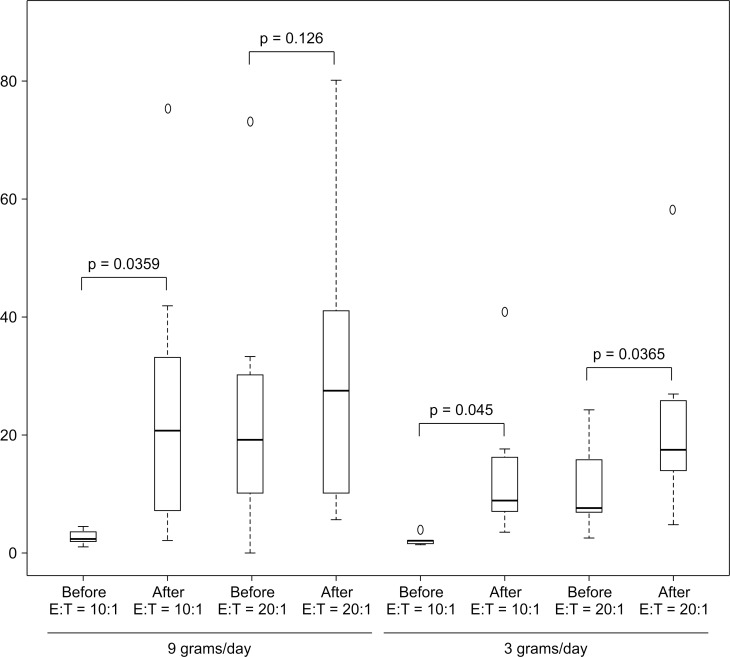
NK cell cytotoxicity induced by FPP administration for 30 days. It has been reported that cytokines and chemokines released in response to lipopolysaccharide (LPS) from the cell membrane of gram-negative bacteria stimulate NK cell activity through dendritic cell (DC) toll-like receptors (TLRs) of intestinal epithelium [48–53]; however, administration of FPP did not affect the level of cytokines or chemokines analyzed in the present study (Table 2).

Administration of FPP at 3 and 9 g/day at an E: T ratio of 10:1 significantly increased NK cell cytotoxicity, whereas administration of FPP at an E: T ratio of 20:1 tended to increase the activity, although it did not reach significance. However, FPP administration at these doses did not affect IgG, IgA, and IgM levels (Table 1, Fig 1).

**Table 1:** PBMC cytolytic activity (NK cell activity) induced by FPP administration for 30 days*.

	PBMC cytolytic activity [10:1 (8.9–29.5), 20:1 (17.1–48.7)]			IgG (820–1740)			IgA (90–400)			IgM [♂(31–200), ♀(52–270)]		
	before	after	*P* value	before	After	*P*	before	after	*P*	before	After	*P*
9 g/day				2017±682	2119±827 ±±827	0.19	360±82	364±103	0.73	123±100	120±86	0.69
E:T = 10:1	2.9±1.1	25.0±16.6	0.035									
E:T = 20:1	24.2±22.6	30.6±25.1	0.126									
3 g/day				1731±506	1697±512	0.30	454±189	436±196	0.09	106±67	101±60	0.07
E:T = 10:1	2.2±0.7	14.4±12.7	0.045									
E:T = 20:1	11.6±8.3	23.0±17.3	0.036									
Control	No FPP	for 30 days		1551±198	1533±280	0.81	500±197	506±207	0.81	74±9	64±12	0.01
E:T = 10:1	2.7±0.9	7.4±4.9	0.088									
E:T = 20:1	10.0±7.1	13.8±7.9	0.256									

*Data in parentheses are normal values for 20–65-year-old healthy subjects.

**Table 2:** Changes in cytokines and chemokines following FPP administration for 30 days*.

	IL-2 (<5)		IL-6 (<6)		IL-10 (<8)		INF-γ (<7.8)		TNF-α (<2.8)	
	before	after	before	after	before	after	before	after	Before	after
9 g/day	all cases <5	all cases <5	all cases <6	all cases <6	all cases <8	all cases <8	all cases <7.8	all cases <7.8	2.16±0.7	2.89±1.2
*P* value		ns		ns		ns		ns		0.98
3 g/day	all cases <5	all cases <5	all cases <6	all cases <6	all cases <8	all cases <8	all cases <7.8	all cases <7.8	2.17±0.7	2.17±0.8
*P* value		ns		ns		ns		ns		0.97

*Data in parentheses are normal values for 20–65-year-old healthy subjects. ns: not significant

These results suggest that FPPs directly influence NK cells in the peripheral blood rather than through LPS-activated TLRs on the intestinal epithelium. FPP administration did not alter the levels of lipid metabolite biomarkers (T-Ch, LDL, and HDL) or markers of inflammation (leukocyte count, monocytes, and c-reactive protein (CRP)) (Tables 3 and 4). It is possible, however, that FPP-induced changes were too small to be detected by our methods.

**Table 3:** Changes in lymphocytes, monocytes, and CRP following FPP administration for 30 days*.

	Normal Value range	Before	After	*P* value
Lymphocyte	(18.0–50.0)			
9 g/day		31.9±5.6	32.9±7.3	0.64
3 g/day		42.8±6.3	38.4±7.0	0.040*
control		23.2±1.8	25.0±5.8	0.49
Monocyte	(1.0–8.0)			
9 g/day		5.9±0.7	6.8±1.1	0.07
3 g/day		6.1±1.0	6.7±0.9	0.24
control		5.5±1.3	5.0±1.3	0.80
CRP	(<0.3)			
9 g/day		1.3±0.9	0.9±0.5	0.48
3 g/day		0.5±0.3	0.3±0.1	0.17
control		0.7±0.7	2.1±4.0	0.41

*Data in parentheses are normal values for 20-65-year-old healthy subjects.

*P < 0.05.

**Table 4:** Changes in lipid metabolites following FPP administration for 30 days*.

Lipid metabolites		Before	After	*P* value
T-ch	(150–219)			
9 g/day		178±25	181±26	0.49
3 g/day		174±29	178±26	0.12
HDL	(males 40–80)			
	(females 40–90)			
9 g/day		42±12	45±12	0.16
3 g/day		56±13	60±16	0.06
LDL	(70–139)			
9 g/day		109±15	112±20	0.51
3 g /day		99±22	99±23	0.90
LDL/HDL ratio	(<2)			
9 g/day		2.7±0.8	2.6±0.7	0.79
3 g/day		1.8±0.5	1.7±0.6	0.17
TG	(50–149)			
9 g/day		154±82	127±51	0.12
3 g/day		74±18	78±20	0.11

*Data in parentheses are normal values for 20-65-year-old healthy subjects.

### Chemical Analyses

CE-TOFMS and LC-TOFMS analyses identified seven low-molecular-weight phenolic acids in the FPP (Table 5); we did not detect significant levels of homovanillic and m-phenolic acid in FPP fermented for less than one year (Table 5). In addition to those listed in Table 5, we identified several other types of organic acids such as lactic acid, various amino acids, and nucleic acids.

**Table 5:** List of primary phenolic acids analyzed by CE- and LC-TOFMS.

Phenolic acids	Lot No.						
	060123	070122	080121	090119	100118	110124	120116
2-Hydroxy-4-methylvaleric acid	1.1E-05	9.9E-05	7.4E-05	9.1E-05	1.0E-05	6.7E-05	5.7E-05
m-Hydroxybenzoic acid	6.2E-05	3.7E-05	5.4 E-05	5.5E-05	6.2E-05	8.8E-05	2.7E-05
2,5-Dihydroxybenzoic acid	5.7E-04	3.0E-04	4.8E-04	5.1E-04	6.6E-04	8.8E-04	2.6E-04
Shikimic acid	4.9E-06	5.2E-06	7.7E-06	6.3E-06	1.2E-05	8.6E-06	7.1E-06
Hippuric acid	1.4E-05	1.3E-05	1.3E-05	1.9E-05	1.5E-05	1.5E-05	1.1E-05
Homovanillic acid	4.7E-05	3.0E-05	3.9E-05	3.0E-05	4.2E-05	4.8E-05	N.D.
Quinic acid	1.0E-04	6.2E-05	6.4E-05	5.2E-05	7.5E-05	1.1E-04	1.6E-04
m-Aminophenol	6.1 E-05	3.7E-05	3.5E-05	3.2E-05	2.5E-05	2.5E-05	N.D.

The first two numbers included in the Lot No. indicate the production year (e.g., 060123 is January 23, 2006).

Numbers in the table indicate relative peak area of phenolic acids.

E: exponential, ND: not detected

### Composition of Fecal Microbiota

**Fig 2:**
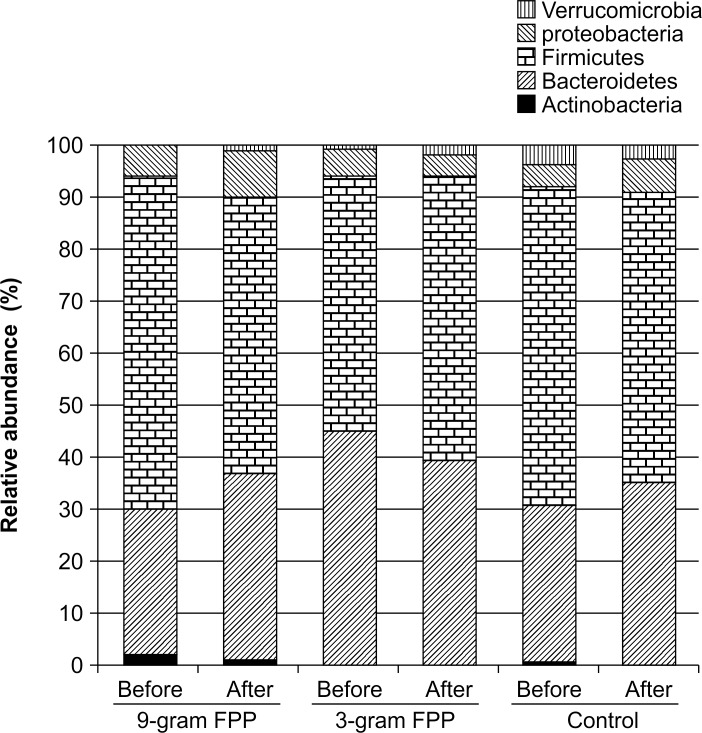
Change in bacterial composition at the phylum level following FPP administration for 30 days. Stool samples were collected before and after the administration of FPP. Bacterial compositions were analyzed by pyrotag sequencing of 16S rRNA genes. The average relative abundance of OTUs for each phylum per treatment group is shown.

Analysis of fecal samples in the control group and at baseline in the FPP groups showed characteristic microbiota with a high proportion of phylum Firmicutes (mean = 57.9%, Fig 2) and genus *Parabacteroides* (mean = 21.2%), and with a low proportion of genus *Bifidobacterium* (mean = 0.97%). At baseline (before the administration of FPP), bifidobacteria were detected in only 3 out of the 21 tested subjects.

**Fig 3:**
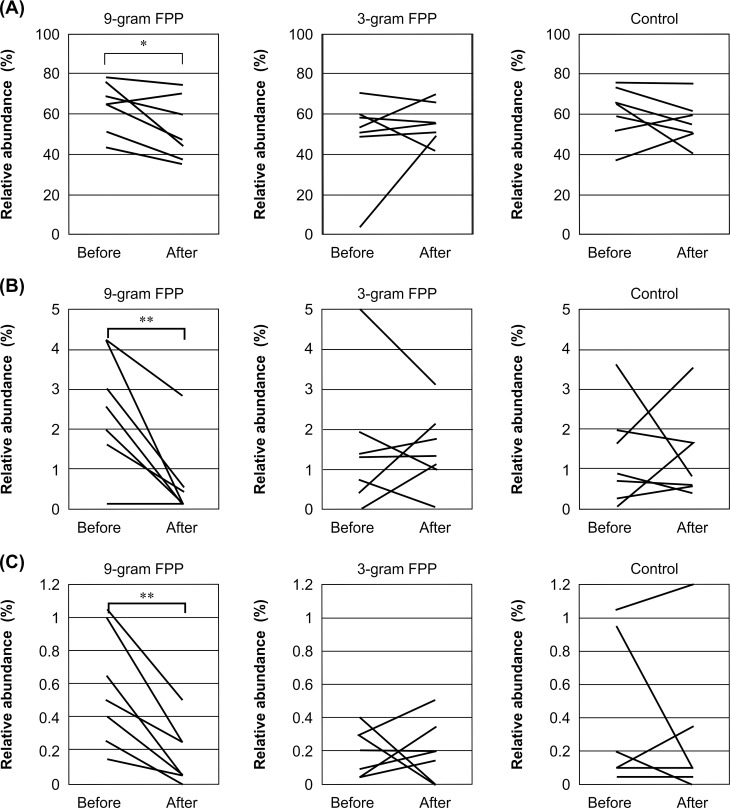
**Change in abundance of Firmicutes (A), OTU308 (*C*. *scindens*) (B), and OTU58 (*E*. *lenta*) (C).** The relative abundance in each sample is plotted. Stool samples were collected before and after the administration of FPP and their bacterial compositions were analyzed by pyrotag sequencing of 16S rRNA genes. Data from the same subject are connected by a line. *P < 0.05 and **P < 0.01 (paired Student’s t-test)

After administration of FPP, the abundance of Firmicutes was significantly decreased (*P* < 0.05, Student’s *t*-test) (Table 6, Figs 2 and 3A). In particular, administration of FPP at 9 g/day reduced the level of OTU308, which is closely related to *C*. *scindens* (Fig 3B), in all subjects but one. The levels of OTU58, which is closely related to *E*. *lenta* (Fig 3C), were also reduced after FPP administration. Interestingly, FPP administration decreased the offensive fecal odor.

**Table 6:** Effects of FPP on fecal microbiota.

Taxonomy	Overall average	FPP at 9 g/day			FPP at 3 g/day			Control group		
		before	after	*P* value	before	after	*P* value	before	after	*P* value
Phylum										
Firmicutes	55.09	63.72	52.96	0.05	49.13	55.32	0.11	50.89	55.94	0.34
Proteobacteria	6.23	5.19	8.43	0.06	5.14	3.33	0.43	4.32	5.66	0.13
Class										
4Cod-2	0.24	0.05	0.36	0.091	0.11	0.34	0.170	0.02	0.56	0.349
Clostridia	49.30	56.14	47.03	0.067	42.60	46.87	0.461	53.46	49.68	0.394
Bacilli	1.51	1.91	0.54	0.229	0.54	0.34	0.094	2.89	2.86	0.936
Family										
Coriobacteriaceae	1.00	0.66	0.21	0.003*	0.61	0.76	0.310	2.32	1.43	0.251
Enterococcaceae	1.08	1.89	0.16	0.096	0.34	0.26	0.245	1.44	2.41	0.356
Porphyromonadaceae	20.39	15.46	23.77	0.089	33.56	22.56	0.177	14.73	12.46	0.546
Ruminococcaceae	13.08	14.92	11.55	0.100	11.43	13.83	0.120	14.29	12.46	0.167
Veillonellaceae	1.95	1.15	1.14	0.095	2.02	2.66	0.240	1.63	2.10	0.338
Genus										
*Anaerofustis*	0.01	0.02	0.00	0.078	0.00	0.01	0.356	0.04	0.00	0.172
*Anaerotruncus*	0.26	0.16	0.16	0.923	0.33	0.61	0.057	0.16	0.16	1.000
*Bilophila*	0.16	0.10	0.24	0.066	0.06	0.06	1.000	0.17	0.31	0.416
*Blautia*	7.16	8.74	3.82	0.015*	7.41	6.17	0.205	11.87	4.96	0.102
*Eggerthella*	0.33	0.60	0.20	0.004*	0.22	0.22	1.000	0.41	0.30	0.434
*Enterococcus*	1.08	1.89	0.16	0.096	0.34	0.26	0.245	1.44	2.41	0.356
*Parabacteroides*	20.34	15.46	23.77	0.089	33.35	22.54	0.177	14.67	12.04	0.518

Taxonomic groups showing significant (*P* < 0.1) population changes after 30-day administration of FPP are listed.

*P < 0.05.

Bacteria in FPPs were analyzed by high-throughput sequencing of amplified 16S rRNA gene fragments. No living bacteria (e.g., *E*. *faecalis* and *A*. *oryzae* used for fermentation) were found in the FPP incubation medium.

## Discussion

The tube fed patients included in this study have chronic inflammatory diseases in addition to their primary diagnoses, as indicated by laboratory inflammatory biomarkers such as elevated CRPs (Table 3).

Many reports have demonstrated the anti-inflammatory effects of several polyphenols [16,17]. Most studies analyzing polyphenols are *in vitro* studies, with limited research in animals and only a few studies in humans. Therefore, many questions about the clinical effects of polyphenols remain unanswered. In fact, there is no information regarding the *in situ* chemical structure, effective dosage, absorption, and metabolism of most FPPs. To our knowledge, the present study is the first to evaluate the clinical effects of FPPs in tube-fed patients.

### Effect of FPP on Immunity

Clinical laboratory effects of FPP administration revealed changes in PBMC cytolytic activity only, which are primarily dependent on the function of NK cell cytotoxicity (as discussed in Materials and Methods) and on the microbiota in feces. Here, we consider PBMC cytolytic activity to be representative of the effects of FPP on NK cell cytotoxicity.

Our results indicate that before FPP administration, NK cell cytotoxicity (cytolytic activity) was lower (E: T = 10:1, 2.56 ± 9.0; 20:1, 24.95 ±15.3) in elderly patients (67–97 years of age) than healthy controls aged 20–60 years (E: T = 10:1, 8.9 ~ 29.5; 20:1, 171 ~ 47.8) (Table 1). NK cell cytotoxicity increased after administration of FPP in a dose-dependent manner, although the effect was not statistically significant because of differences between strong and poor responders.

It is reported that the number of NK cells increases and their components change (e.g., CD56 increases) in the elderly; however, NK cell cytotoxicity decreases [54–56]. Decreased NK cell cytotoxicity in the elderly is not dependent on NK cells, but rather on the host environment [57]; however, the decrease observed in this study was not restored by IL-15/IL-15R mediated by IL-2 [57,58]. These experiments suggest that the decrease in NK cell cytotoxicity may be caused by the environment surrounding the aged NK cells and/or intracellular events such as energy production and signal trafficking, rather than by the NK cells themselves. NK cells are activated in the presence of LPS released from probiotics, which is accompanied by changing cytokines and chemokines. LPS stimulates TLR-4 in colon membrane DCs and enhances the expression of interferon-γ (INF-γ) and tumor necrosis factor-α (TNF-α), which in turn stimulates NK cell surface receptors [59–61]. In our study, however, NK cell activity was enhanced by FPPs without upregulation of IL-2, IL-6, IL-10, INF-γ, or TNF-α (Tables 1–3) (Fig 1). In addition, there were no observed increases in gram-negative bacteria (*Lactococcus* and *Bifidobacteroides*), which are found in probiotics and reported to be stimulators of TLR-4, or living bacteria (*E*. *faecalis* and *A*. *oryzae* used for fermentation) in FPP, as determined by 16S rRNA analysis of the incubation media.

These results suggest that FPP do not increase NK cell cytotoxicity by activating cytokines. Studies have shown that polyphenols (epigalocatechin gallate, oenothelin B, ellagitanin, and resveratrol) augment NK cell cytotoxicity [62–65]; however, a study of hesperidin showed no effects in healthy, well-nourished humans [62]. In an *in vitro* study, INF-γ concentrations were increased after treatment with resveratrol; however, because the study was done in a cell culture system, release of INF-γ was likely the result of NK cell activation [66]. While treatment with FPP augmented NK cell cytotoxicity in elderly patients, it is possible that it has no effect on NK cell cytotoxicity in healthy young people, as observed for hesperidin [62].

Further studies are needed to determine which low-molecular-weight phenolic acids, phenolic acid combinations, or ratios are most effective for activating NK cells without activating cytokines and chemokines or increasing the frequency of NK cells.

### Molecular Structure/function Relationship, Absorption, and Effective Dosage

Bioactive polyphenols are polymerized large molecules and not easily absorbed from digestive tracts as their original large molecules; therefore, FPP bioactivity, molecular structure/function relationship, and absorption are important considerations. There are many reports of *in vivo* and *in vitro* experiments showing that low-molecular-weight phenolic acids have various bioactivities, including antioxidant activity [25–29]; however, these studies do not indicate the bioactive molecular structure/functional relationships.

FPP contains single phenolic acids and many kinds of low-molecular-weight substances, but no polymerized structures; however, these low-molecular-weight substances behave similar to polymerized polyphenols *in vivo*.

There are relatively few reports that studied the relationship between the molecular structure and biological activity of polyphenols as whole a molecule [66–68]. Park *et al*. [67] reported differences between monomeric, dimeric, and trimeric flavonoids in nitric oxide (NO) production, TNF-α secretion, and NF-κB-dependent gene expression in RAW 254.7 macrophages. These responses were repressed by monomers and dimers, but enhanced by trimers. However, these experiments were conducted *in vitro*, and further *in vivo* studies are needed to confirm these findings in animals and humans.

Williamson *et al*. [68] suggested that colonic catabolites of orally administered polyphenols are the “missing” compounds, and that they are potentially important compounds that mediate some of the biological activities and health benefits of polyphenol-rich foods.

The chemical structure and effective dosage of functional candidate compounds are different *in vivo* from that *in vitro*. The majority of polyphenols are broken down and absorbed in the intestines by intestinal microflora, suggesting that the original structures of polyphenols do not necessarily mediate their functions *in vivo*.

The level of polyphenols such as procyanidins, chlorogenic acids, and anthocyanins are lower in peripheral blood than that of other flavonoids, even after administration of high doses or consumption of large amounts of foods rich in these compounds [69]. In this regard, several studies have investigated the absorption and metabolism of certain flavonoids such as quercetin and (-)-epicatechin [62]. Intervention studies involving consumption of procyanidin-, chlorogenic acid-, or anthocyanidin-rich foods have shown that these foods do not change the levels of certain biomarkers [70,71]. However, these results could be explained by the low blood concentrations of parent compounds and their failure to affect the levels of these biomarkers [72,73]. Based on the effective doses *in vivo*, these reports also indicated that the effects of polymerized polyphenols do not necessarily resemble those of the original chemical structure.

Polyphenols are primarily absorbed from the large intestine (90–95%), although some are absorbed from the small intestine (5–10%) [74–87]. Interestingly, the concentration of excreted polyphenols in urine and feces is less than the amount ingested [72]. This fact indicates that a large percentage of consumed polyphenols is metabolized to small molecules and absorbed; the metabolites are then consumed in biological reactions.

These studies suggest that the low-molecular-weight phenolic acids in FPP have the same bioactivities as the original polymerized polyphenols in *C*. *papaya* L.

### Fermentation of Polyphenols

*C*. *papaya* L. is fermented under aerobic conditions; aerobic metabolites are different from those formed under anaerobic conditions, such as in the colon. The polyphenol catabolites of aerobic fermentation exert better biological effects than the polyphenols in grapes [85]. Several studies have demonstrated that fermentation enhances polyphenol bioactivity [86–88], suggesting that fermentation results in the conversion of polyphenols with large molecular weights to compounds of low molecular weight, which have more biological activity. Under aerobic conditions where fermentation is influenced by oxygen, oxidized structures are generated, giving rise to lower-molecular-weight compounds that are converted into mono-aromatic acids and CO_2_ [89].

### Prebiotic Effects of FPP on Colonic Flora

The type and amount of food consumed daily, such as volume of fiber, may explain the profile of fecal microflora of FPP. Daily recommendations in Japan (version 2010) include consumption of more than 17 g/day of dietary fiber. Our tube-fed patients receiving FPP were supplied with approximately 17 g/day of fiber. Commercially available tube nutrition includes an average of 1.5 g fiber/100 kcal/100 g and provide from 800 to 1,000 kcal. Therefore, dietary fiber deficiencies are not likely to be the reason for the characteristic features of fecal microflora described in this study. FPP did not increase *Bifidobacterium* or decrease Bacteroidetes, but significantly reduced the abundance of Firmicutes, particularly the class *Clostridia*, including *E*. *lenta* and *C*. *scindens* (Table 6). These results suggest that bile acids are decreased, as supported by a previous study showing the outgrowth of *Clostridia* in rats fed high concentrations of bile acids [90].

Polyphenols and/or their catabolites could alter the composition of gut microflora by reducing the colonic pH value, suppressing Bacteroidetes and pathogenic *Clostridium perfringens* and *Clostridium difficile*, and increasing the proportion of *Bifidobacteria* and eubacteria without inhibiting lactic acid bacteria [91,92]. Gallic acid and caffeic acid have been reported to repress *Clostridium* and *Bacteroides* species [93]. These effects were also observed in our study, although the exact mechanisms are not yet clear. Our results indicate that low-molecular-weight phenolic acids in FPP affect the composition of colon microflora similarly to polymerized polyphenols.

The (C_6_-C_1_) low-molecular-weight polyphenol catabolites (e.g., protocatechuic acid) reduce serum levels of total cholesterol, LDL, and HDL in overloaded rats [94,95]. Data from animal and *in vitro* studies suggest that (C_6_-C_2_) and especially (C_6_-C_3_) [96,97] catabolites interfere with various enzymes in the mevalonate pathway. For example, 3-hydroxy-3-methylglutaryl-CoA reductase reduced glucose levels in an experimental type II diabetes animal model [98,99]. These experiments were performed in overloaded animals and rats with genetically induced diabetes. Our study, however, showed no changes in lipid metabolism after administration of FPP. Elderly tube-fed patients are often discouraged from consuming cholesterol-rich and fat-rich nutrients or excessive calories. Fig 2 indicates that tube-fed patients are typically supplied with the same kind of artificial nutrition over long periods without supplementary natural probiotic foods or are treated frequently with antibiotics against repeated infections. Our results suggest that FPP and probiotic supplementation is useful for tube-fed patients to maintain a healthy immune status.

### Conclusions

Our results show that older patients who are tube-fed over the long term have decreased NK cell toxicity, and that low-molecular-weight phenolic acids produced by the fermentation of polymerized large molecular weight polyphenols are bioactive. In addition, these low-molecular-weight phenolic acids exert their effects, such as increasing NK cell activity, without causing hypercytokinemia, hyperchemokinemia, or changing the components of the gut microbiota.

The present study does have limitations. For example, we did not classify NK cell frequency, which may further explain our results showing enhanced NK cell cytotoxicity. *C*. *papaya* L. is not the only source of polyphenols, with many polymerized large molecular weight polyphenols existing in the plant world; however, most of them are difficult to digest or absorb. Consequently, development of fermentation biotechnology of polymerized large molecular weight polyphenols may introduce useful low-molecular-weight phenolic acids with improved absorption and human health benefits.
